# The Physician’s Library

**Published:** 1894-12

**Authors:** Edward Cranch

**Affiliations:** Erie, Pa., Class of ’75


### THE PHYSICIAN'S LIBRARY.

Edward Cranch, M. D., Erie, Pa., Class of '75.

Books are clearly indispensable, for reference and for study, to help in shaping a well-equipped mind—one that is ready when confronted by work to do, one that knows how to record old facts and new truths, and from these, as from fruitful seeds, propagate newer truths that shall bring forth fruit in future fields of use.

It remains to consider what sort of books are essential and what desirable. Beware of buying long rows of books to be used only in "future leisure," a deceitful will-o'-the-wisp that often lures one to many a needless purchase. Be warned in time!

Anatomy must have its text-book and its atlas, the latter to have handy when patients want enlightenment, a point which most appreciate very highly. Henke's is a good one, McClellan's fuller and more exact.

Physiology has yet to have its ideal manual, but its study in lectures is so fascinating that it is rarely forgotten, and doubtless for that reason seldom consulted. If you get one let it be bound in cloth, for old editions rapidly lose all value.

Chemistry needs ready and exact reference, such as in the works of Bowman, Wolff, Galloway, and Attfield; but urinology and toxicology must be inscribed upon the living mind indelibly.

Next in logical order comes materia medica, in which department we deem this much at least absolutely essential: an encyclopedic work, such as Allen's or Hering's or both; a shorter manual, such as Jahr's or Hering's condensed; and an old-school work, such as the *National Dispensatory* or Shoemaker's *Materia Medica.*

But consider no such work superfluous; this branch should be as complete as possible, and as rapidly as you can pray acquire Hahnemann's *Materia Medica* and *Chronic Diseases,* Lippe, Guernsey, Farrington, Dunham, Allen's *Handbook* and *Primer,* Hughes, the *Pathogenesy,* Teste, Hoyne, McMichael, Dewey, Jones, Hale, and the rest, and study them in their own light and by the light of *The Organon* till they mean something to you, and till you *think* you can write one for yourself. Get the repertories, including the *Bee Line,* and make yourself familiar with them.

Next comes diagnosis, mostly only supplementary to the clinical instruction at the college, without which books of this class are of no more value than anatomy without a full course of dissections.

Works on practice, such as Raue's, Pepper's, Flint's, and Ziemssen's, generally contain enough diagnosis and pathology for early reference.

As a guide to therapeutics, Hahnemann's *Organon,* stripped of its bitter invective is super-eminent, and should be read in the original, in the fourth and fifth editions, and in Dudgeon's translation or Stratten's, but the latter is long out of print.

Next in value comes Jahr's *Forty Years' Practice*, which ought to be re-edited in pocket form, and which is also the best book for a domestic guide, if one must be furnished. Next come Lilienthal's *Therapeutics*, Minton's *Uterine Diseases*, which includes as pretty a *résumé* of materia medica as is to be found anywhere. Allen on *Intermittent Fever*, of which a similar remark is in order, only there are not so many drugs, Bell on *Diarrhœa*, Wells on *Dysentery and Diarrhœa*, the *Twelve Tissue Remedies*, and a host of valuable monographs and larger works.

Surgery needs Helmuth and his followers, with perhaps an old-school work for contrast, but not many, for these, like physiology, biology, and histology go rapidly to new editions. Do not despise minor surgery, for a reputation is often made or lost in this field. Know your "bones" like an "end man," but don't rattle them unless you have to.

Obstetrics and gynæcology are just now enjoying a new birth, and you must ask your professors; we confess to bewilderment in face of the hosts. Only do not forget these two adages: "Fear the man of one book," and again, "He who reads many books on one subject cuts the widest swath." Know at least one book thoroughly, but read many.

Hygiene, bacteriology, histology, and climatology must have a corner, at least for thought, while a good jurisprudence and *The Physician Himself*, by Cathell, may help to warn you of pitfalls in the path.

Get one of Miner's combined day-book and ledger for your accounts, and study it well. Take notes of *every case*, carrying a little pad or blank-book, and filing the sheets in an office file. You will regret it if you do not; such a habit promotes exact prescribing and wins patients.

Do not omit at least one or two journals, and borrow all the rest you can read. Belong to at least one society and contribute to its transactions and preserve them. Then with all these you will have a good working library, and can add dictionaries, special works, collateral works, and all the rest you choose.—*The Chironian.*

